# Kimura or Warshaw? Development and Validation of a Preoperative Prediction Model Based on 3D Reconstruction Technology

**DOI:** 10.1245/s10434-025-18548-3

**Published:** 2025-10-13

**Authors:** Hongliang Liu, Qisheng Hao, Lunan Wu, Mengxing Cheng, Fabo Qiu, Chuandong Sun, Hao Zou, Bin Zhou

**Affiliations:** 1 Department of Hepatobiliary and Pancreatic Surgery and Retroperitoneal Tumor Surgery, The Affiliated Hospital of Qingdao University, Qingdao, China; 2 Department of Thoracic Surgery, Women and Children’s Hospital Affiliated to Qingdao University, Qingdao, China

**Keywords:** Kimura, Warshaw, Splenic vessel preservation, Three-dimensional reconstruction, Nomogram, Prediction model

## Abstract

**Background:**

The purpose of this study was to investigate the risk factors for splenic vessel preservation in laparoscopic Kimura procedure and to establish and validate a nomogram model for predicting the failure of splenic vessel preservation.

**Patients and Methods:**

Clinical data of 169 patients who underwent spleen-preserving distal pancreatectomy for benign or low-grade malignant tumors of the distal pancreas at the Affiliated Hospital of Qingdao University from July 2018 to April 2024 were retrospectively collected. Among them, there were 55 male and 114 female individuals, with an average age of 48.24 ± 14.62 years. All patients were planned to undergo laparoscopic Kimura procedure on the basis of preoperative discussions. A total of 118 patients (69.82%) were randomly assigned to the modeling group, and 51 patients (30.18%) to the validation group. No significant differences were observed in baseline characteristics between the two groups (all *P* > 0.05). On the basis of data from the modeling group, independent risk factors for splenic vessel preservation were identified using rank sum test and multivariate logistic regression analysis. R software was used to establish a nomogram model for predicting the failure of splenic vessel preservation, and external validation was performed using data from the validation group.

**Results:**

On the basis of data from the modeling group, the cutoff values of tumor volume, ratio of splenic vein (SV) circumference embedded in pancreas, and pancreas–SV contact area were determined using receiver operating characteristic curves as follows: 9828.8 mm^3^ [area under the curve (AUC) = 0.745, 95% confidence interval (CI) 0.654–0.835], 0.45 (AUC = 0.751, 95% CI 0.610–0.893), and 501 mm^2^ (AUC = 0.715, 95% CI 0.588–0.903), respectively. The AUC for tumor location was 0.733 (95% CI 0.622–0.874). Univariate and multivariate logistic regression analyses showed that tumor volume, tumor location, ratio of SV circumference embedded in pancreas, and pancreas–SV contact area were valuable preoperative indicators for predicting the failure of splenic vessel preservation (*P* < 0.05). The nomogram prediction model established using R software on the basis of these four indicators exhibited good predictive performance, with C-indices of 0.747 in the modeling group and 0.756 in the validation group. Decision curve analysis indicated that the nomogram model had favorable clinical benefits.

**Conclusions:**

Tumor volume, tumor location, ratio of SV circumference embedded in pancreas, and pancreas–SV contact area are valuable preoperative indicators for predicting the risk of splenic vessel preservation failure. The nomogram model established on the basis of these indicators has certain clinical practical value. As a representative of the integration of medicine and engineering, three-dimensional medical reconstruction can provide an important reference for the selection of surgical procedures in laparoscopic spleen-preserving distal pancreatectomy.

In recent years, with the continuous development of the concept of precise surgery and laparoscopic minimally invasive surgery, there has been a growing trend toward individualized spleen-preserving distal pancreatectomy for benign or low-grade malignant tumors of the pancreatic body and tail.^1^ As early as 1988, Warshaw^2^ first reported spleen-preserving distal pancreatectomy with splenic vessel resection. This technique made it possible to preserve the spleen in patients with splenic vessel involvement. However, this procedure involves cutting off the splenic artery and vein, which is prone to two major complications: splenic infarction and gastric varices. From the perspective of evidence-based medicine, therefore, spleen-preserving distal pancreatectomy with preservation of the splenic artery (SA) and vein should be advocated. In 1996, Kimura et al.^3^ reported distal pancreatectomy with preservation of the spleen and splenic vessels. This procedure fully preserves the blood supply to the spleen and significantly reduces the incidence of splenic infarction. However, it requires dissection of the splenic artery and vein, which increases the difficulty of the operation and makes intraoperative injury to the splenic vessels likely, leading to massive bleeding.^4^ Therefore, to accurately assess the difficulty of the operation before surgery, select the appropriate surgical method, and reduce the occurrence of intraoperative accidents, it is necessary to establish a relatively accurate clinical prediction model on the basis of preoperative indicators to guide clinicians in making correct surgical choices. By retrospectively analyzing the general conditions, serological values, pathology-related indicators, and three-dimensional medical reconstruction-related indicators of patients scheduled to undergo the Kimura procedure preoperatively, we established a visual risk prediction model for splenic vessel preservation and evaluated the predictive performance and clinical practicality of the model through internal and external validation.

## Patients and Methods

### Study Subjects

Inclusion criteria were as follows: (1) pathologically diagnosed as benign or low-grade malignant tumor of the pancreatic body and tail; (2) planned to undergo Kimura procedure as documented in preoperative discussion records; and (3) successfully completed laparoscopic spleen-preserving distal pancreatectomy. Exclusion criteria were as follows: (1) incomplete clinical data; (2) failure to complete three-dimensional reconstruction; and (3) history of previous pancreatic surgery.

Clinical data of patients who underwent spleen-preserving distal pancreatectomy for benign or low-grade malignant tumors of the pancreatic body and tail at the Affiliated Hospital of Qingdao University from July 2018 to April 2024 were retrospectively collected. A total of 169 patients were included in the study according to the inclusion and exclusion criteria, including 55 male and 114 female individuals, with an age of 48.24 ± 14.62 years.

A total of 118 patients (69.82%) were randomly assigned to the modeling group and 51 (30.18%) to the validation group. No significant differences were observed in baseline characteristics between the two groups (all *P* > 0.05). The former was used to establish the nomogram model, and the latter to verify the reliability of the model. Comparisons of general data between the two groups are presented in Table 1. This study conforms to the requirements of the Declaration of Helsinki, and all patients signed informed consent forms before surgery.

**Table 1:** Comparison of general data between the modeling group and the validation group

Factor	Modeling group (*n* = 118)	Validation group (*n* = 51)	Statistic	*P*
Age (years), M (Q_1_, Q_3_)	47 (35.75, 57)	49 (36, 64)	*Z* = −0.41	0.68
BMI (kg/m^2^), M (Q_1_, Q_3_)	24.9 (23.42, 26.75)	24.7 (23.4, 26.6)	*Z* = −0.40	0.69
Gender, *n* (%)			*χ*^2^ = 0.86	0.35
Male	41 (34.7)	14 (27.5)		
Female	77 (65.3)	37 (72.5)		
History of hepatitis B, *n* (%)			*χ*^2^ = 0.00	0.99
None	104 (88.1)	45 (88.2)		
Yes	14 (11.9)	6 (11.8)		
Cirrhosis, *n* (%)			*χ*^2^ = 0.000	1.00
None	109 (92.4)	47 (92.2)		
Yes	9 (7.6)	4 (7.8)		
Smoking history, *n* (%)			*χ*^2^ = 0.01	0.94
None	109 (92.4)	48 (94.1)		
Yes	9 (7.6)	3(5.9)		
Drinking history, *n* (%)			*χ*^2^ = 0.00	1.00
None	108 (91.5)	47 (92.2)		
Yes	10 (8.5)	4 (7.8)		
Diabetes, *n* (%)			*χ*^2^ = 0.80	0.37
None	107 (90.7)	49 (96.1)		
Yes	11 (9.3)	2 (3.9)		
History of pancreatitis, *n* (%)			*χ*^2^ = 0.02	0.90
None	111 (94.1)	47 (92.2)		
Yes	7 (5.9)	4 (7.8)		
History of abdominal surgery, *n* (%)			*χ*^2^ = 0.01	0.95
None	113 (95.8)	48 (94.1)		
Yes	5 (4.2)	3 (5.9)		
White blood cell count (× 10^9^/L), M (Q_1_, Q_3_)	5.52 (4.63, 6.70)	5.83 (4.56, 6.7)	*Z* = −0.20	0.84
Neutrophil ratio (%), M (Q_1_, Q_3_)	55 (49.90, 62.28)	53.3 (49.6, 64.3)	*Z* = −0.02	0.98
Hemoglobin (g/L), M (Q_1_Q_3_)	133.5 (125.5, 141)	132 (123, 141)	*Z* = −0.46	0.64
Total bilirubin (μmol/L), M (Q_1_, Q_3_)	12.75 (10.24, 15.8)	13.23 (10.79, 15.8)	*Z* = −0.46	0.65
Albumin (g/L), M (Q_1_, Q_3_)	42.22 (40.08, 44.88)	42.6 (40.1, 45.5)	*Z* = −0.46	0.65
HDL (mmol/L), mean ± SD	1.38 ± 0.29	1.41 ± 0.31	*t* = −0.62	0.54
LDL (mmol/L), mean ± SD	3.03 ± 0.89	3.02 ± 0.85	*t* = 0.06	0.95
Platelet (× 10^9^/L), mean ± SD	233.09 ± 52.48	234.67 ± 52.32	*t* = −0.18	0.86
ASA score, *n* (%)			*χ*^2^ = 0.21	0.65
Grade 1	101 (85.6)	45 (88.2)		
Grade 2	17 (14.4)	6 (11.8)		
Tumor location, *n* (%)			*χ*^2^ = 0.33	0.57
Body	97 (82.2)	40 (78.4)		
Tail	21 (17.8)	11 (21.6)		
Tumor volume (mm^3^), M (Q_1_, Q_3_)	8093.33 (3315.65, 23,012.83)	7621.30 (4255.30, 30,439.42)	*Z* = −0.17	0.94
TG (mmol/L), M (Q_1_, Q_3_)	1 (0.79, 1.39)	0.94 (0.67, 1.36)	Z = −0.86	0.39
TC (mmol/L), M (Q_1_Q_3_)	4.97 (4.17, 5.71)	5 (4.26, 5.71)	*Z* = −0.41	0.68
Maximum tumor diameter (cm), M (Q_1_Q_3_	3 (2.2, 4)	2.8 (2.2, 4)	*Z* = −0.13	0.89
Ratio of SV length embedded in pancreas, M (Q_1_, Q_3_)	0.21(0.12, 0.32)	0.22 (0.15, 0.32)	*Z* = −0.58	0.56
Ratio of splenic vein (SV) circumference embedded in pancreas, M (Q_1_, Q_3_)	0.19 (0.15, 0.30)	0.2 (0.15, 0.28)	*Z* = −0.32	0.75
Pancreas–SV contact area (mm^2^), M (Q_1_, Q_3_)	269.76 (206.88, 460.47)	297 (208.96, 402.54)	*Z* = −0.17	0.87
Minimum distance between tumor and blood vessel (mm), M (Q_1_, Q_3_)	1.42 (0.71, 5.4)	1.11 (0.61, 2.91)	*Z* = −0.82	0.41
Ratio of SA length embedded in pancreas, M (Q_1_, Q_3_)	0.16 (0.09, 0.22)	0.14 (0.08, 0.22)	*Z* = −0.05	0.96
Ratio of SA circumference embedded in pancreas, M (Q_1_, Q_3_)	0.13 (0.07, 0.15)	0.12 (0.08, 0.15)	*Z* = −0.02	0.99
Pancreas–SA contact area (mm^2^), M (Q_1_, Q_3_)	130.32 (74.13, 184.50)	128.43 (71.26, 174.69)	*Z* = −0.10	0.92
Number of collateral branches of SV on pancreatic surface, M (Q_1_, Q_3_)	3 (2, 4)	4 (2, 5)	*Z* = −0.96	0.34
Number of collateral branches of SA on pancreatic surface, M (Q_1_, Q_3_)	2 (1, 3)	2 (1, 3)	*Z* = −0.12	0.90
Maximum curvature of SV (°), M (Q_1_, Q_3_)	105.84 (93.55, 118.59)	107.86(96.38, 119.96)	*Z* = −0.51	0.61
Tumor type, *n* (%)			*χ*^2^ = 0.59	0.44
Solid	37 (31.4)	13 (25.5)		
Cystic	81 (68.6)	38 (74.5)		

### Data Collection

**Fig. 1:**
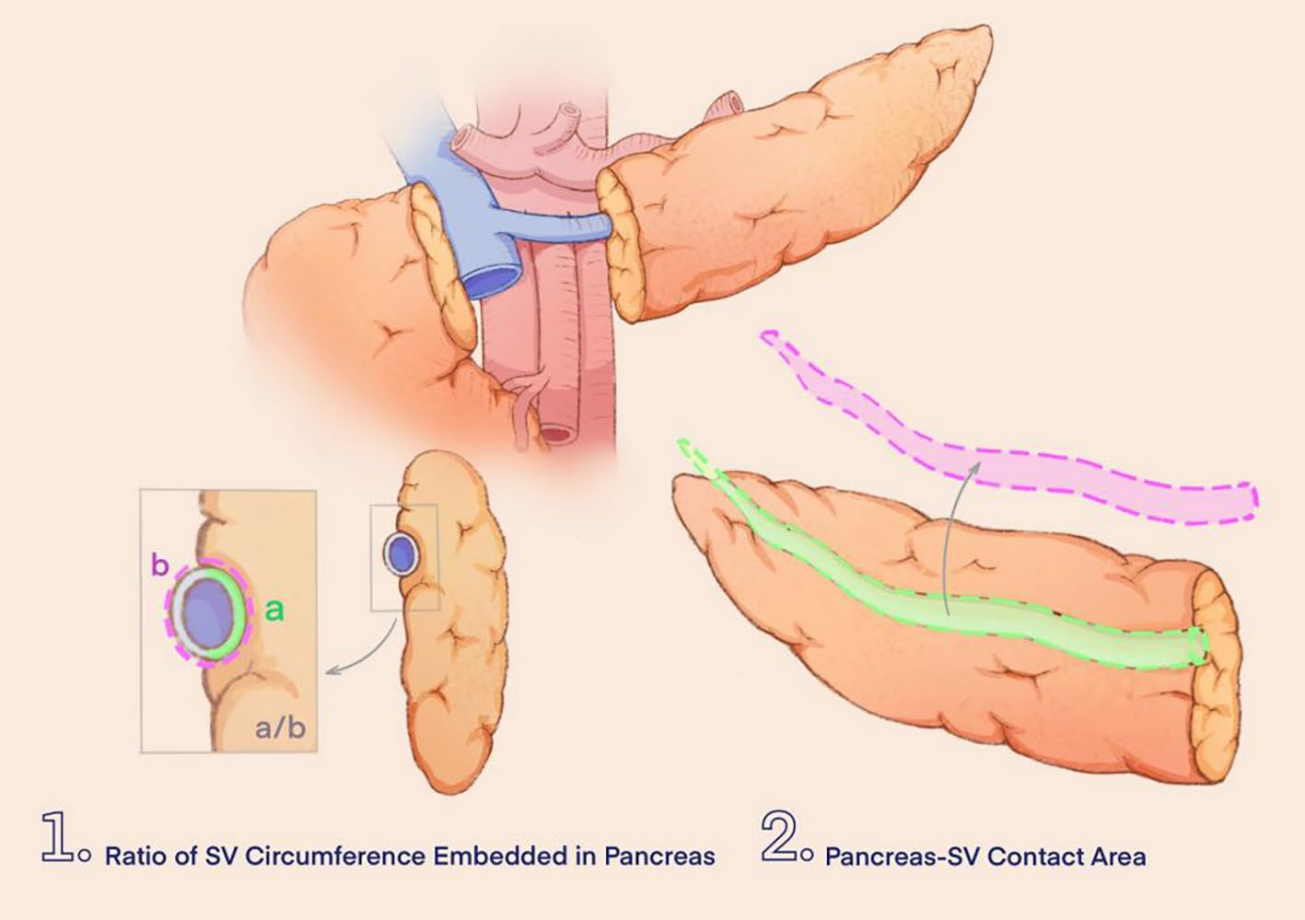
Schematic diagrams of representative reconstruction indicators; (1A/B) circumference of the splenic vein embedded in the pancreas/the total circumference of the splenic vein, (2) total contact area between the resected pancreas and the splenic vein

The data collected included the following: (1) general information of patients: age, gender, body mass index (BMI), history of pancreatitis, history of abdominal surgery, smoking history, drinking history, ASA score, etc.; (2) preoperative serological indicators: white blood cell count, platelet level, liver function-related indicators, blood lipid indicators, etc.; (3) pathology-related indicators: tumor type, location of pancreatic tumor, maximum diameter of tumor, etc. ; (4) three-dimensional (3D) medical reconstruction-related indicators: tumor volume, ratio of SV circumference embedded in pancreas, pancreas–SV contact area, number of collateral branches of the SV on the pancreatic surface, curvature of the SV, etc. Schematic diagrams of representative reconstruction indicators are shown in Fig. 1.

Data completeness was high for all collected variables. There were no missing data for the clinical, pathological, or 3D reconstruction parameters used in the final analysis. This was ensured by the study’s inclusion criteria, which mandated successful 3D reconstruction and the availability of complete clinical records. As such, no imputation methods for missing data were necessary.

### Three-Dimensional Reconstruction and Engineering Calculation

**Fig. 2:**
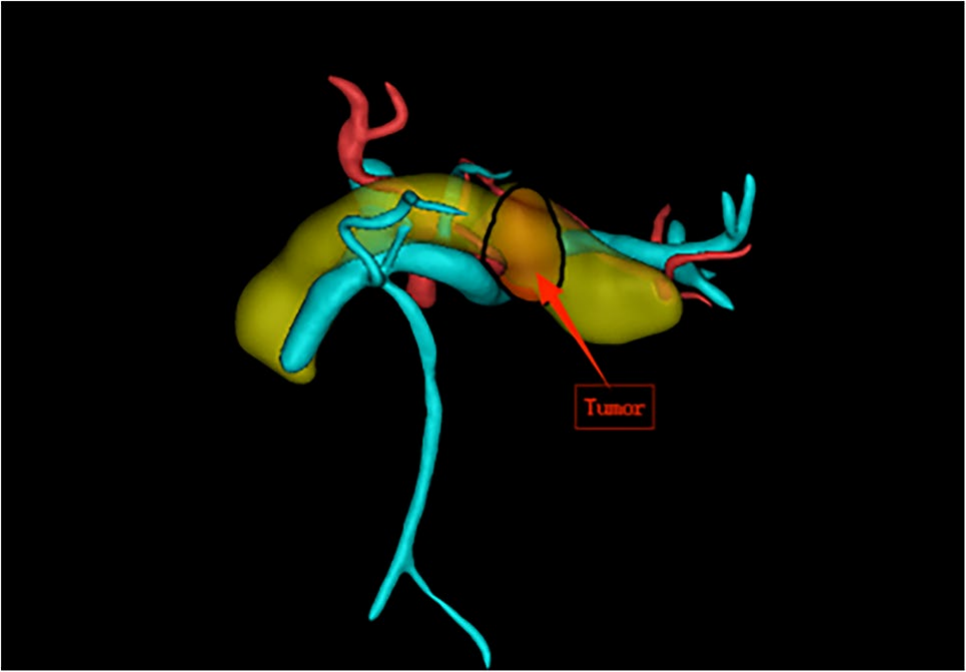
Front view example of three-dimensional reconstruction

**Fig. 3:**
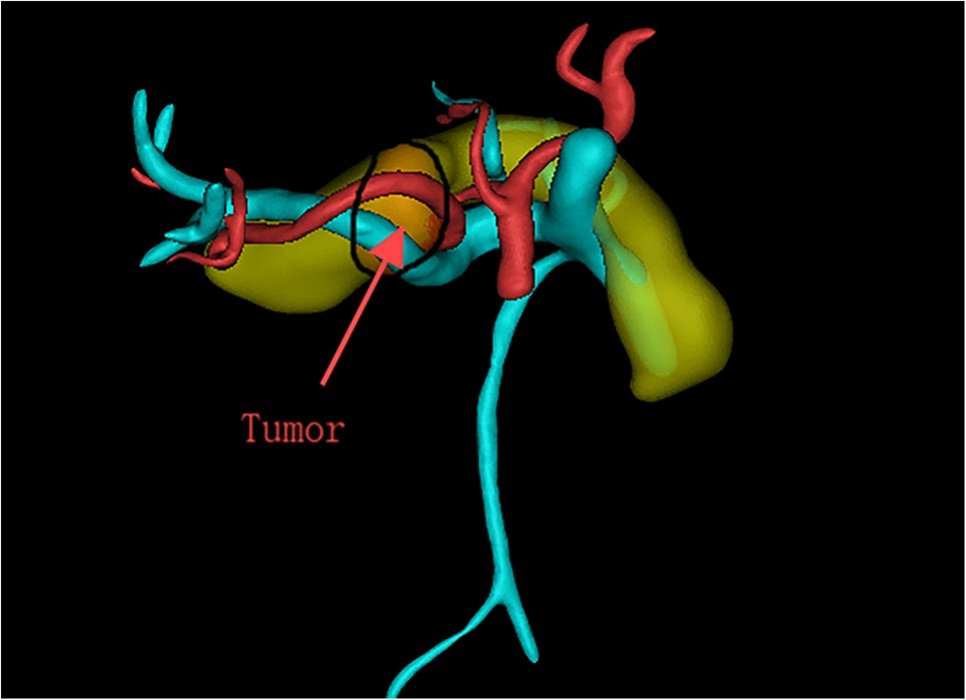
Rear view example of three-dimensional reconstruction

**Fig. 4:**
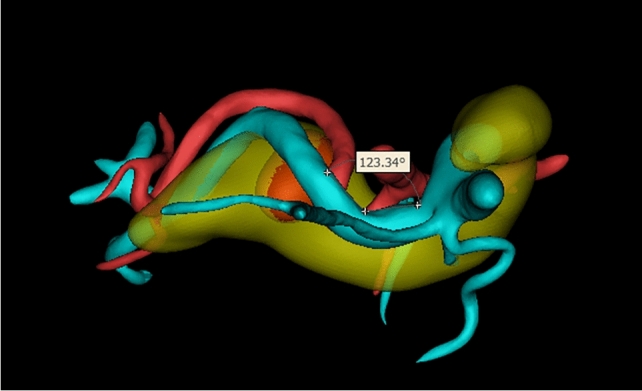
Example of three-dimensional reconstruction measurement indicators (maximum curvature of the SV)

**Fig. 5:**
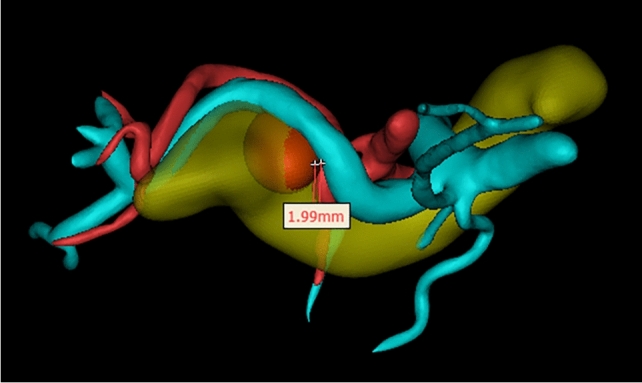
Example of three-dimensional reconstruction measurement indicators (shortest distance between tumor edge and blood vessel)

**Fig. 6:**
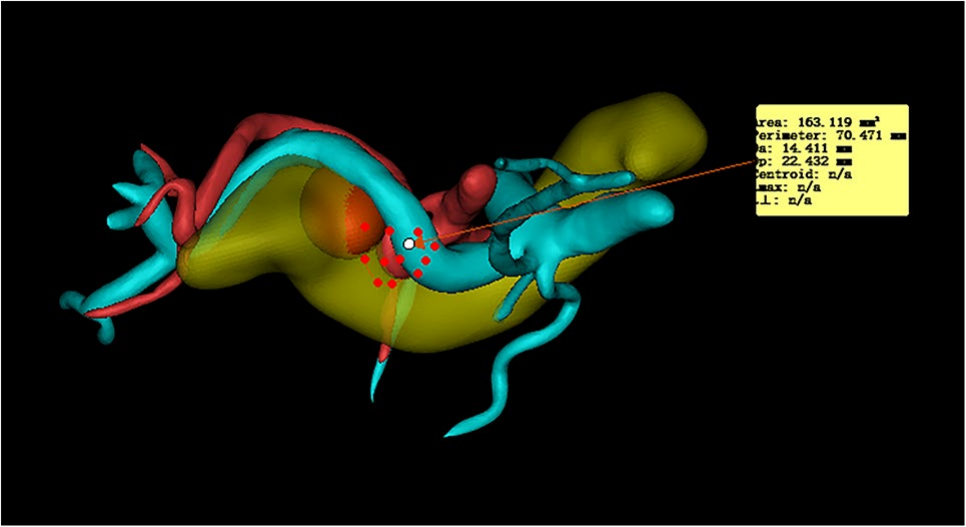
Example of three-dimensional reconstruction measurement indicators (contact area between SV and pancreas)

Mimics 21.0 was used for three-dimensional reconstruction of pancreatic arteries, veins, and pancreatic tumors. Firstly, computed tomography/magnetic resonance imaging (CT/MRI) images were imported, and two-dimensional structures of pancreatic arteries, veins, and tumors were extracted through threshold segmentation and region growing, followed by three-dimensional calculation to generate corresponding three-dimensional models. Then, feature points of arteries, veins, and tumors were selected in the three-dimensional view to measure relevant indicators. After completing vessel segmentation, click the “measure” menu and select the “distance/angle/perimeter” tool. Click on the contours of the segmented SV or SA in sequence; the software will automatically calculate and display the perimeter values of the vessels in the measurement result window. For the SV and SA, measurements should be performed separately, with corresponding data carefully recorded. Click the “tools” menu and select the “centerline extraction” option. In the pop-up “centerline extraction” dialog box, select the segmented region of the SV as the input object, then set appropriate parameters such as sampling interval and smoothing factor. Generally, the sampling interval can be set to 1–2 mm, and the smoothing factor to 0.5–1.0; specific values can be adjusted according to vessel thickness and image quality. Click the “OK” button, and the software will generate the centerline of the SV, representing the approximate direction of the vessel. The “measure angle” function under “measure” was used to select two points on the vessel for measuring curvature. After completing tumor segmentation, click the “measure” menu and select the “volume” option. The software will automatically calculate and display the volume value of the segmented pancreatic tumor in the measurement result window, with the unit typically being cubic millimeters (mm^3^). Click the “view” menu and select the “multi-planar reconstruction (MPR)” function. By adjusting the positions and angles of the three MPR planes (coronal, sagittal, and transverse), the optimal view for clearly displaying the contact surface between the pancreas and splenic artery/vein (i.e., the pancreatic surface) was determined. The plane position could be precisely adjusted by dragging sliders or inputting coordinate values, while observing the image to ensure the pancreatic surface appears as a continuous and clear plane for observing and counting collateral vessels. Representative three-dimensional reconstruction images are shown in Figs. 2, 3, 4, 5 and 6.

### Surgical Procedure

For all cases, we first attempted to preserve the splenic vessels using the Kimura technique. After general anesthesia with endotracheal intubation, the patient was placed in a supine lithotomy position with the head elevated and feet lowered, and the camera holder stood between the patient’s legs. A five-port approach was adopted: a 10-mm arc incision at the lower edge of the umbilicus was made as the observation port, and a trocar was inserted to establish pneumoperitoneum; the other four ports were distributed in a V shape. Routine abdominal exploration was performed to rule out tumor metastasis. The gastrocolic ligament was opened with an ultrasonic scalpel to expose the pancreas. A blunt instrument was used to lift the gastric body upward, fully revealing the pancreatic body and tail. The lower edge of the pancreas was dissected with an ultrasonic scalpel to expose the SV, and the space between the pancreas and SV was freed; subsequently, the SA was exposed, and the space between the lower end of the pancreas and the SA was carefully dissected. After freeing a 3–5 cm segment of the pancreatic body from the splenic vessels, the pancreas was transected 2–3 cm proximal to the tumor using a laparoscopic linear cutting stapler (Endo-GIA). Dissection of the space between the distal pancreatic body–tail and the splenic vessels was continued. Arterial branches or venous tributaries entering the pancreatic body–tail were clipped with absorbable clips or Hem-o-lock clips before transection. For bleeding, 5-0 Prolene sutures were used for ligation to achieve hemostasis until complete resection of the pancreatic body and tail. If splenic vessel injury was irreparable or bleeding was uncontrollable, splenic vessel preservation was deemed impossible, and the procedure was converted to the Warshaw technique. The proximal splenic artery and vein were clipped and transected, and the distal splenic vessels were transected using a vascular stapler.

### Statistical Methods

SPSS 25.0 software was used for statistical analysis. The Shapiro–Wilk test (W-test) was applied to verify the normality of data distribution. Normally distributed quantitative data were expressed as x ± s, and intergroup differences were tested using the independent samples *t*-test. Non-normally distributed quantitative data were expressed as M (Q_1_, Q_3_), and intergroup differences were tested using the rank-sum test. Categorical data were expressed as counts (percentages), and intergroup differences were tested using the chi-squared (*χ*^2^) test. The rank-sum test was used to screen indicators with intergroup differences, and receiver operating characteristic (ROC) curves were employed to determine the cutoff values of each indicator for predicting the risk of splenic vessel preservation failure. Multivariate logistic regression analysis was performed to identify independent risk factors for splenic vessel preservation failure, which were then used to establish the nomogram prediction model. The rms package in R software was used to draw the nomogram. To internally validate the nomogram and correct for overfitting (optimism), bootstrap validation was performed. Specifically, we generated 10,000 bootstrap samples by randomly sampling with replacement from the original modeling cohort (*n* = 118). For each sample, a new model was refitted and its performance was evaluated both on the bootstrap sample and on the original cohort. The average difference in performance (optimism) was subtracted from the apparent performance of the original model to obtain an optimism-corrected C-index. Additionally, the rms and rmda packages were used to perform decision curve analysis (DCA). A *P*-value < 0.05 was considered statistically significant.

## Results

### Determination of Relevant Indicators and Their Cutoff Values in the Modeling Group

In the modeling group, splenic vessels were successfully preserved in 98 cases, while preservation failed in 20 cases. Statistically significant differences were observed between the two groups in terms of tumor volume, tumor location, ratio of SV circumference embedded in pancreas, and pancreas–SV contact area (all* P* < 0.05); no statistically significant differences were found in other indicators between the two groups (all *P* > 0.05). Details are presented in Table 2.

**Table 2:** Comparison of relevant indicators between the successful splenic vessel preservation group and the failed splenic vessel preservation group in the modeling group

Factor	Successful splenic vessel preservation group (*n* = 98)	Failed splenic vessel preservation group (*n* = 20)	Statistic	*P*
Age (years), M (Q_1_, Q_3_)	47 (35, 56.25)	48 (40, 65.75)	Z = −0.73	0.46
BMI (kg/m^2^), M (Q_1_, Q_3_)	24.90 (23.50, 26.75)	24.31 (21.74, 27.67)	Z = −0.65	0.51
Gender, *n* (%)			*χ*^2^ = 0.001	0.98
Male	34 (34.7)	7 (35)		
Female	64 (65.3)	13 (65)		
History of hepatitis B, *n* (%)			*χ*^2^ = 0.73	0.39
None	88 (89.8)	16 (80)		
Yes	10 (10.2)	4 (20)		
Cirrhosis, *n* (%)			*χ*^2^ = 0.00	1.00
None	91 (92.9)	18 (90)		
Yes	7 (7.1)	2 (10)		
Smoking history, *n* (%)			*χ*^2^ = 0.001	0.98
None	90 (91.8)	19 (95)		
Yes	8 (8.2)	1 (5)		
Drinking history,* n* (%)			*χ*^2^ = 0.00	1.00
None	90 (91.8)	18 (90)		
Yes	8 (8.2)	2 (10)		
Diabetes, *n* (%)			*χ*^2^ = 0.29	0.59
None	90 (91.8)	17 (85)		
Yes	8 (8.2)	3 (15)		
History of pancreatitis, *n* (%)			*χ*^2^ = 0.00	1.00
None	92 (93.9)	19 (95)		
Yes	6 (6.1)	1 (5)		
History of abdominal surgery, *n* (%)			*χ*^2^ = 0.00	1.00
None	94 (95.9)	19 (95)		
Yes	4 (4.1)	1 (5)		
White blood cell count (× 10^9^/L), M (Q_1_, Q_3_)	5.53 (4.58, 6.66)	5.34 (4.76, 6.79)	*Z* = −0.48	0.63
Neutrophil ratio (%), M (Q_1_, Q_3_)	55(49.55, 62.40)	55.80 (50.58, 62.67)	*Z* = −0.50	0.62
Hemoglobin (g/L), M (Q_1_, Q_3_)	132 (126.75, 141)	138 (121.50, 143.75)	*Z* = −0.47	0.64
Total bilirubin (μmol/L), M (Q_1_, Q_3_)	12.79 (10.24, 15.91)	12.46 (9.61, 15.16)	*Z* = −0.63	0.53
Albumin (g/L), M (Q_1_, Q_3_)	42.22 (39.83, 44.54)	42.19 (40.64, 45.30)	*Z* = −0.32	0.75
HDL (mmol/L), mean ± SD	1.37 ± 0.29	1.41 ± 0.30	*t* = −0.51	0.61
LDL (mmol/L), mean ± SD	3.05 ± 0.93	2.95 ± 0.65	*t* = 0.46	0.65
Platelet (× 10^9^/L), mean ± SD	230.49 ± 50.89	245.85 ± 59.42	*t* = −1.20	0.23
ASA score, *n* (%)			*χ*^2^ = 0.00	1.00
Grade 1	84 (85.7)	17 (85)		
Grade 2	14 (14.3)	3 (15)		
Tumor location, *n* (%)			*χ*^2^ = 49.26	< 0.05
Body	92 (93.9)	5 (25)		
Tail	6 (6.1)	15 (75)		
Tumor volume (mm^3^), M (Q_1_, Q_3_)	5996.42 (2950.52, 12,677.75)	22,545.140 (12,633.86, 53,972.33)	*Z* = −5.62	< 0.05
TG (mmol/L), M (Q_1_, Q_3_)	0.96 (0.75, 1.39)	1.16 (0.94, 1.51)	*Z* = −1.56	0.12
TC (mmol/L), M (Q_1_, Q_3_)	5 (4.20, 5.71)	4.74 (4.04, 5.42)	*Z* = −0.66	0.51
Maximum tumor diameter (cm), M (Q_1_, Q_3_)	3 (2, 4)	3 (2.50, 4.53)	*Z* = −1.30	0.19
Ratio of SV length embedded in pancreas, M (Q_1_, Q_3_	0.20 (0.14, 0.31)	0.18 (0.13, 0.24)	*Z* = −0.50	0.62
Ratio of SV circumference embedded in pancreas, M (Q_1_, Q_3_)	0.24 (0.08, 0.44)	0.38 (0.31, 0.58)	*Z* = −5.81	<0.05
Pancreas–SV contact area (mm^2^), mean ± SD	272.40 ± 156.91	693.89 ± 374.37	*t* = −4.95	<0.05
Minimum distance between tumor and blood vessel (mm), M (Q_1_, Q_3_)	1.66 (0.72, 5.84)	0.91 (0.68, 1.53)	*Z* = −1.35	0.18
Ratio of SA length embedded in pancreas, M (Q_1_, Q_3_)	0.16 (0.09, 0.23)	0.17 (0.08, 0.23)	*Z* = −0.05	0.96
Ratio of SA circumference embedded in pancreas, M (Q_1_, Q_3_)	0.12 (0.08, 0.15)	0.14 (0.06, 0.15)	*Z* = −0.21	0.84
Pancreas–SA contact area (mm^2^), M (Q_1_, Q_3_)	131.80 (79.29, 186.06)	107.29 (67.44, 164.69)	*Z* = −1.11	0.27
Number of collateral branches of SV on pancreatic surface, M (Q_1_, Q_3_)	3 (2, 4)	3.5 (2, 4)	*Z* = 0.00	1.00
Number of collateral branches of SA on pancreatic surface, M (Q_1_, Q_3_)	2 (1, 3)	2 (1, 3.75)	*Z* = −1.22	0.22
Maximum curvature of SV (°), M (Q_1_, Q_3_)	107.86 (95.75, 119.26)	99.93 (90.96, 113.32)	*Z* = −1.32	0.19
Tumor type, *n* (%)			*χ*^2^ = 0.02	0.89
Solid	31 (31.6)	6 (30.0)		
Cystic	67 (68.4)	14 (70.0)		

**Fig. 7:**
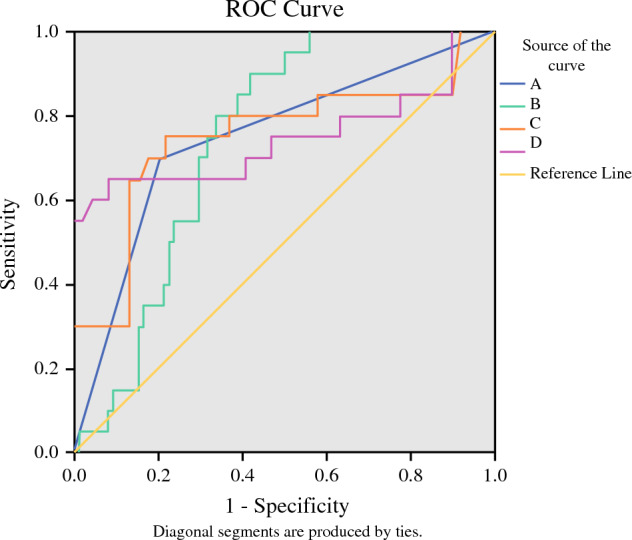
Receiver operating characteristic curves of four indicators for predicting splenic vessel preservation failure: **A** tumor location, **B** tumor volume, **C** ratio of SV circumference embedded in pancreas, and **D** pancreas–SV contact area

Cutoff values for indicators with intergroup differences in predicting splenic vessel preservation failure were determined using ROC curves. The area under the curve (AUC) for tumor location was 0.733 (95% CI 0.622–0.874). The cutoff value for tumor volume was 9828.8 (AUC = 0.745, 95% CI 0.654–0.835), the cutoff value for ratio of SV circumference embedded in pancreas was 0.45 (AUC = 0.751, 95% CI 0.610–0.893), and the cutoff value for pancreas–SV contact area was 501 (AUC = 0.715, 95% CI 0.588–0.903), as shown in Fig. 7.

### Independent Risk Factors or Splenic Vessel Preservation Failure

The results of multivariate logistic regression analysis showed that tumor volume, tumor location, Ratio of SV Circumference Embedded in Pancreas, and Pancreas–SV Contact Area were independent risk factors for splenic vessel preservation failure in spleen-preserving distal pancreatectomy (all *P* values < 0.05), as presented in Table 3.

**Table 3:** Results of multivariate logistic regression analysis for factors associated with splenic vessel preservation

Factor	*β*	SEM	*Wald*	95% CI	*P*
Tumor location	3.181	1.424	4.992	1.478–3.846	0.025
Tumor volume	0.000	0.000	5.737	0.980–1.000	0.017
Ratio of SV circumference embedded in pancreas	−11.567	4.134	7.830	0.010–0.031	0.005
Pancreas–SV contact area	−0.005	0.002	4.182	0.991–1.000	0.041

### Establishment and Validation of the Nomogram Model

**Fig. 8:**
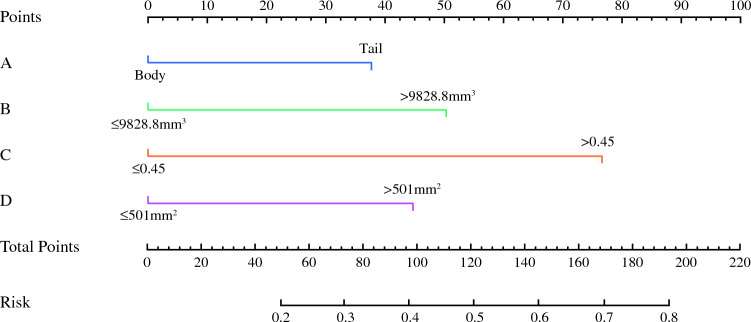
Nomogram model for predicting the failure of splenic vessel preservation using four indicators: **A** tumor location, **B** tumor volume, **C** ratio of SV circumference embedded in pancreas, and **D** pancreas–SV contact area

**Fig. 9:**
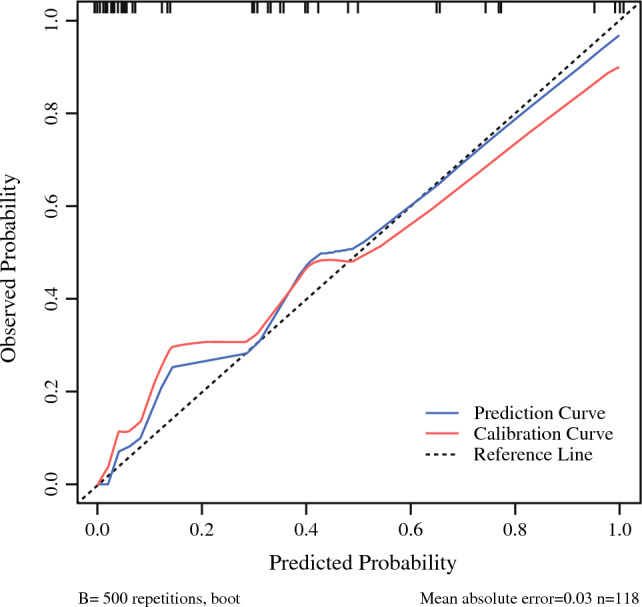
Calibration curve of the nomogram prediction model for preoperative prediction of the risk of splenic vessel preservation failure

**Fig. 10:**
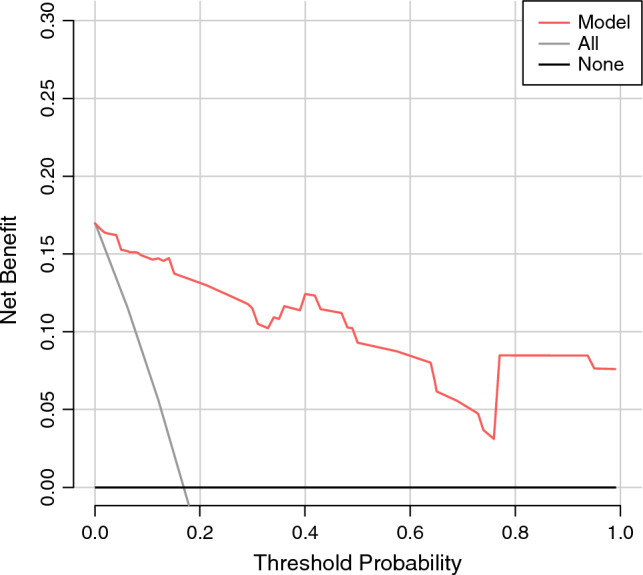
Clinical decision curve of the nomogram model for preoperative prediction of the risk of splenic vessel preservation failure

**Fig. 11:**
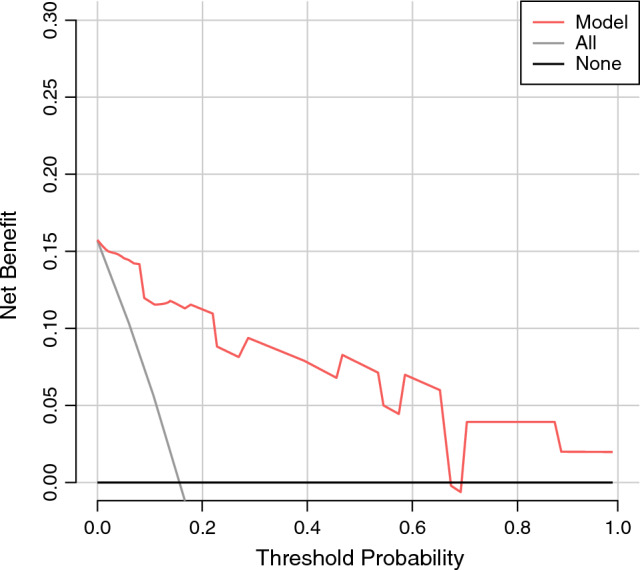
Clinical decision curve analysis of patients undergoing spleen-preserving distal pancreatectomy in the validation cohort

A nomogram model consisting of the above independent risk factors was established (Fig. 8). The value of each risk factor corresponds to a specific score, and the total score is converted via the nomogram into the probability of splenic vessel preservation failure during spleen-preserving distal pancreatectomy. The C-index of the nomogram model was 0.747 (95% CI 0.698–0.820). Internal validation via 10,000 repeated samplings using the bootstrap method yielded a C-index of 0.721 (> 0.7), indicating good consistency between the model’s predicted results and actual outcomes. The calibration curve showed that both the nomogram’s prediction curve and the calibrated prediction curve were close to the ideal result, demonstrating favorable predictive performance (Fig. 9). Validation using data from the validation group revealed an area under the ROC curve of 0.756 (95% CI 0.596–0.878), indicating good discriminative ability of the model. Decision curve analysis (DCA) of the nomogram model confirmed its favorable clinical benefits (Figs. 10, 11).

## Discussion

Spleen-preserving distal pancreatectomy can be classified into two techniques, namely Kimura and Warshaw on the basis of whether the splenic vessels are preserved during the operation. The key and challenge of the Kimura technique lie in the complete separation of the splenic vessels, particularly the SV, from the pancreatic body and tail. In contrast, the Warshaw technique bypasses the need for such vascular dissection, resulting in a relatively lower surgical difficulty. If a patient is not suitable for splenic vessel preservation but the clinician insists on performing the Kimura procedure, it may easily lead to uncontrollable intraoperative massive bleeding. Conversely, if a patient is eligible for splenic vessel preservation but the clinician opts for the Warshaw technique to minimize risks, it may increase the incidence of postoperative complications such as splenic infarction.^5^ Therefore, preoperative assessment of tumor location and its relationship with blood vessels is of paramount importance. Currently, preoperative evaluation is mostly conducted using CT, MR, and other imaging modalities combined with clinicians’ experience, lacking precise quantitative methods for assessing surgical risks.^6^ Studies have demonstrated that preoperative three-dimensional (3D) visual surgical simulation can improve the success rate and safety of laparoscopic distal pancreatectomy with spleen preservation.^7^ Our research center has upgraded the 3D reconstruction system and integrated it with engineering calculation technology, achieving a more accurate assessment of vascular anatomy and the relationship between blood vessels and tumors, which provided strong technical support for the smooth implementation of this study.

In the conclusions of this study, tumor volume is identified as one of the independent risk factors affecting splenic vessel preservation—the larger the tumor volume, the higher the risk of failure in preserving the splenic vessels. This is consistent with the findings of previous related studies.^8–11^ Dai et al.^12^ found that when the tumor size is 3 cm, the sensitivity and specificity for predicting the need for Warshaw surgery are 82.0% and 60.4%, respectively. In the study by Xu et al.,^13^ with a tumor volume cutoff value of 2.5 cm, the sensitivity and specificity for predicting the Warshaw procedure are 96.55% and 65.52%, respectively. In our study, the cutoff value of tumor volume was 9828.8 mm^3^, with a sensitivity and specificity of 90.02% and 58.26% for predicting the failure of splenic vessel preservation, respectively. These results are generally consistent with the aforementioned studies. However, it is evident that the sensitivity and specificity of using tumor size alone to predict the feasibility of splenic vessel preservation remain unsatisfactory. It is certain that for benign tumors of the pancreatic body and tail, despite their low invasiveness, larger tumors are often difficult to mobilize, thereby increasing surgical complexity. Additionally, larger tumors exert stronger compressive effects; when surrounding organs and vessels are compressed, aseptic inflammation may occur, and the infiltration of inflammatory cells further complicates the dissection of splenic vessels. Furthermore, tumors located in the pancreatic tail are also a risk factor for splenic vessel preservation failure. Compared with tumors in the pancreatic body, tail tumors are closer to the splenic hilum, where the risks of vascular variations and adhesions are higher, increasing surgical complexity. Moreover, tail tumors tend to induce more inflammation and vascular compression, thereby escalating the difficulty of splenic vessel dissection.

All patients in this study successfully underwent spleen-preserving distal pancreatectomy. The primary reasons for failure to preserve the splenic vessels were uncontrollable bleeding from the SV (*n* = 22) and dense adhesions between the tumor/pancreas and the splenic vessels (*n* = 6). Regarding the SA, it typically runs along the superior margin of the pancreas with a certain gap between them, and it is enclosed in an arterial sheath, allowing traction with a vascular sling. Thus, dissecting the branches of the SA in the pancreatic body and tail is relatively easier. In contrast, the SV courses deep on the posterior surface of the pancreas, and a portion of it may be embedded within the pancreatic parenchyma. Particularly in patients with larger pancreatic tumors, the SV may be compressed and densely adherent to the tumor, making dissection of the SV and its branches more challenging and often leading to massive bleeding. Therefore, in this study, we incorporated several engineering measurement indices related to blood vessels, including the ratio of SA circumference embedded in pancreas, ratio of SV circumference embedded in pancreas, pancreas–SV contact area, pancreas–SA contact area, number of collateral branches of the SV on the pancreatic surface, and the curvature of the SV. Data analysis ultimately identified the ratio of SV circumference embedded in pancreas and pancreas–SV contact area as independent risk factors for splenic vessel preservation failure. These two indices effectively quantify the correlation between the SV and the pancreas. The “pancreas-SV contact area” was chosen as a metric over a linear “contact length” as it provides a superior, three-dimensional representation of the interface between the tumor and the vessel. This area more accurately captures the surgical challenge of mobilizing the splenic vein from a broad, adherent surface along its curvature. A higher ratio of the circumference of the SV embedded in the pancreas to its total circumference indicates a more severe encasement of the SV by the pancreas, rendering dissection more difficult. A larger contact area between the SV and the pancreas increases the difficulty of separation, requiring more effort from surgeons during dissection and elevating the risks of intraoperative bleeding and failed vascular separation.

We established and validated a nomogram with clinical benefits on the basis of four indices (Fig. 8). To translate the nomogram into a practical tool for preoperative planning, we propose the following evidence-based guidance. On the basis of the predicted probability of splenic vessel preservation failure, patients can be stratified into three management categories. Firstly, for patients with a low risk (< 20%), the Kimura procedure should be confidently attempted, as the benefits of preserving splenic vascularity outweigh the minimal risk of conversion. Secondly, for those in the intermediate risk range (20–50%), a prudent approach is recommended. Surgeons should initiate the operation with the intention of performing the Kimura technique but must be fully prepared for the possibility of conversion to the Warshaw method. This necessitates having vascular control equipment readily available and ensuring the anesthesia team is prepared for potential blood loss. Finally, for patients identified as high risk (≥ 50%), directly proceeding to the Warshaw technique is strongly advised. This threshold is clinically intuitive, as a predicted failure rate of more than half signifies that attempting vessel preservation is more likely to lead to uncontrolled bleeding and prolonged operation than success. This recommendation is further supported by our model output (Figure 8), which demonstrates that a total score exceeding approximately 130 points—often resulting from the presence of three risk factors—reaches this probability threshold. By adopting this stratified approach, surgeons can move from a reactive to a proactive strategy, optimizing patient safety and operational efficiency.

In summary, on the basis of the clinical model developed through the integration of medicine and engineering in this study, the Kimura technique can be attempted for patients with a low risk of splenic vessel preservation failure to retain the splenic vessels, while the Warshaw technique can be directly adopted for high-risk patients. This model enables surgeons to transition from a passive to an active role, allowing them to predict risks in advance and proactively select the surgical approach. It transforms the traditional “operating while thinking” model, reduces intraoperative accidental injury and bleeding of the splenic vessels, and avoids passive splenectomy. Preoperative surgical strategies can be formulated to develop individualized treatment plans, thereby further improving the success rate of spleen preservation, making the surgery more precise and minimally invasive, and ultimately benefiting patients. However, this study has several limitations: (1) this study is retrospective in nature, which may introduce selection bias despite our efforts to include all eligible patients meeting the inclusion criteria. Although we employed strict inclusion and exclusion criteria and randomized the cohort into modeling and validation groups to mitigate bias, unmeasured confounding factors may still exist. Additionally, the sample size of our single-center validation cohort (*n* = 51), while adequate for initial validation, is relatively limited. This may affect the precision of the model’s performance estimates. Therefore, the results of our external validation should be interpreted as preliminary. Future multicenter, prospective studies with larger sample sizes are necessary to further validate and refine this prediction model before it can be widely recommended for clinical use. (2) While 3D reconstruction technology provides valuable anatomical insights, it has certain limitations. The model is based on static imaging data and cannot dynamically simulate tissue elasticity, adhesion density, or intraoperative deformation. Moreover, the accuracy of measurements may be influenced by image resolution and segmentation subjectivity. Future integration with real-time surgical navigation or elastic modeling may further enhance preoperative planning. (3) The experience, skills, and subjectivity of the operating surgeon also influence the success rate of splenic vessel preservation to a certain extent.

## Data Availability

The datasets analyzed during the current study are available from the corresponding author on reasonable request.
